# Alterations of plasma exosomal proteins and motabolies are associated with the progression of castration-resistant prostate cancer

**DOI:** 10.1186/s12967-022-03860-3

**Published:** 2023-01-21

**Authors:** Pengyu Liu, Wenxuan Wang, Fei Wang, Jiaqi Fan, Jinan Guo, Tao Wu, Dongliang Lu, Qingchun Zhou, Zhuohao Liu, Yuliang Wang, Zhiqun Shang, Franky Leung Chan, Wei Yang, Xin Li, Shan-Chao Zhao, Qingyou Zheng, Fei Wang, Dinglan Wu

**Affiliations:** 1 Shenzhen Key Laboratory of Viral Oncology, The Clinical Innovation & Research Center (CIRC), Shenzhen Hospital, Southern Medical University, Shenzhen, Guangdong Province China; 2 Department of Urology, Hainan General Hospital, Hainan Affiliated Hospital of Hainan Medical University, Haikou, Hainan Province China; 3 Department of Urology, Shenzhen People’s Hospital (The Second Clinical Medical College, Jinan University, The First Affiliated Hospital, Southern University of Science and Technology), Shenzhen, Guangdong Province China; 4 Department of Urology, Shenzhen Hospital, Southern Medical University, Shenzhen, Guangdong Province China; 5 Department of Urology, Tianjin Institute of Urology, The Second Hospital of Tianjin Medical University, Tianjin, 300211 China; 6 School of Biomedical Sciences, The Chinese University of Hong Kong, Hong Kong, SAR China; 7 Department of Pathology, School of Basic Medical Sciences, Southern Medical University, Guangzhou, 510515 China; 8 Department of Endocrinology and Metabolism, Zhujiang Hospital, Southern Medical University, Guangzhou, 510280 China; 9 Department of Urology, The Third Affiliated Hospital of Southern Medical University, Guangzhou, 510500 China; 10 Department of Urology, Nanfang Hospital, Southern Medical University, Guangzhou, 510515 China; 11 Department of Medical Genetics and Developmental Biology, School of Medicine, Southeast University, Nanjing, China; 12 Department of Urology, Guangdong Hospital of Traditional Chinese Medicine, Zhuhai, Guangdong Province 519015 China

**Keywords:** PCa, CRPC, Exosomes, Proteomics, Metabolomics, Biomarkers

## Abstract

**Background:**

Current diagnosis tools for prostate cancer (PCa) such as serum PSA detection and prostate biopsy cannot distinguish dormant tumors from invasive malignancies, either be used as prognosis marker for castration resistant prostate cancer (CRPC), the lethal stage of PCa patients. Exosomes have been widely investigated as promising biomarkers for various diseases. We aim to characterize the proteomic and metabolomic profile of exosomes and to evaluate their potential value for the diagnosis of PCa, especially CRPC. We also investigate the functions of some specific exosome biomarkers in the progression of CRPC.

**Methods:**

Integrated proteomics and metabolomics analysis were performed for plasma-derived exosomes collected from tumor-free controls (TFC), PCa and CRPC patients. Expression of specific exosomal proteins were further validated by targeted 4D-parallel reaction monitoring (PRM) mass spectrometry among the three cohorts. Tissue distribution and functional role of exosomal protein LRG1 was studied in clinical PCa tissue samples and cell line models.

**Results:**

Three potential exosomal protein markers were identified. The apolipoprotein E level in PCa samples was 1.7-fold higher than that in TFC (receiver operating characteristic value, 0.74). Similarly, the levels of exosome-derived leucine-rich alpha2-glycoprotein 1 (LRG1) and inter-alpha-trypsin inhibitor heavy chain H3 (ITIH3) in the CRPC group were 1.7 and 2.04 times, respectively, higher than those in the PCa group (ROC values, 0.84 and 0.85, respectively), indicating that LRG1 and ITIH3 could serve as predictive markers for CRPC. For metabolomic evaluation of exosomes, a series of differentially expressed metabolites were identified, and a combined metabolite panel showed ROC value of 0.94 for distinguishing PCa from TFC and 0.97 for distinguishing CRPC from PCa. Immunohistochemistry of tissue microarray showed that LRG1 protein was significantly upregulated in advanced prostate cancer and functional assay revealed that ectopic expression of LRG1 can significantly enhance the malignant phenotype of prostate cancer cells. More importantly, PCa cell derived LRG1-overexpressed exosomes remarkably promoted angiogenesis.

**Conclusion:**

Integration of proteomics and metabolomics data generated proteomic and metabolic signatures of plasma exosomes that may facilitate discrimination of CRPC from PCa and TFC patients, suggesting the potential of exosomal proteins and metabolites as CRPC markers. The study also confirmed the important role of exosomal protein LRG1 in PCa malignant progression.

**Supplementary Information:**

The online version contains supplementary material available at 10.1186/s12967-022-03860-3.

## Introduction

Prostate cancer (PCa) is one of the most common malignancies in developed Western countries, with the highest incidence recorded in the United States [1]. Due to population aging, lifestyle changes and the increasing popularization of PCa screening, the incidence and detection rate of PCa in Asian countries has also been increasing gradually [2]. Androgen deprivation therapy (ADT) is still the principal treatment option for locally advanced and metastatic prostate cancers. However, most patients received ADT inevitably develop resistance to treatment and relapse with a more aggressive form of castration-resistant prostate cancer (CRPC) within 2–3 years. At present, there is a lack of highly specific and sensitive molecular markers to precisely predict the progression of PCa to CRPC, and to evaluate the treatment of advanced prostate cancer.

Prostate-specific antigen (PSA) testing have been used for PCa screening for more than 35 years [3]. Although PSA shows moderate sensitivity and specificity, it cannot distinguish between dormant tumors and invasive malignancies and cannot be used to assess the clinical benefit in patients who receive ADT [4, 5]. Moreover, PSA detection cannot be applied to infer the mortality associated with PCa, but instead reflects the trend in PCa incidence worldwide [6]. Therefore, researchers have been attempting to identify novel biomarkers for PCa precise diagnosis. One such potential biomarker, prostate cancer antigen 3 (PCA3), is a prostate-specific non-coding RNA that has been proven to be a promising PCa biomarker and more accurate than traditional PSA evaluations, particularly for patients with ambiguous prostate biopsy results [7]. However, the potential for over detection and overtreatment of indolent tumors on the basis of PCA3 assessments needs to be clarified, because the correlation between PCA3 and long-term overall survival remains debatable [8]. Thus, more accurate evaluation of PCa patients using effective and specific biomarkers are required to facilitate risk-assessment and treatment options, and thereby maximize the benefits for patients.

Exosomes are extracellular vesicles with a phospholipid bilayer structure that are secreted by a variety of cells and carry various biomacromolecules, including nucleic acids, proteins, lipids, and metabolites [9]. These biomacromolecules serve as significant mediators for intercellular communication in physiological and pathological processes. Exosomes have been recently shown to be key drivers of tumor progression and are associated with a series of tumor behaviors, including tumor growth, metastasis, and the tumor microenvironment [10]. Based on their advantages of tumor specificity, non-invasiveness, and rapid detection, exosomal biomarkers have shown extremely high diagnostic value for the evaluation of malignant tumors [11, 12].

Previous studies on tumor-derived exosomes have mainly focused on non-coding RNAs [13]. However, with the rapid advancements in high-sensitivity mass spectrometry, detection of proteins and metabolites using more precise and quantitative approaches has become possible. In particular, proteins derived from exosomes offer unique advantages over traditional serological markers. First, partial exosomal proteins prefer to be released outside specific cells. For instance, the phosphorylated nuclear transcription factor X box binding 1 (NFX1) can be captured only in the exosomes of breast cancer patients, which may suggest that the functions of phosphorylated NFX1 are mediated under specific conditions [14]. Second, tumor-derived exosomes show higher specificity than those obtained from healthy donors. The exosome-derived GPC1 was enriched in pancreatic cancer patients and showed excellent ability in comparison with carbohydrate antigen (CA) 19-9 or serum-free GPC1 in pancreatic cancer screening [15]. Third, the natural barrier effect of lipid bilayers ensures that exosomal proteins are hardly degraded by external proteases and other enzymes. The resultant outstanding stability confers exosomes the potential to serve as the PCa biomarkers [14].

Metabolic abnormalities have been widely characterized as a distinguishing feature of tumors [16]. Metabolic changes are closely related to disease progression and directly reflect the tumor microenvironment, cellular status, and clinical drug response [17]. Thus, in comparison with transcriptomics and proteomics, analyses based on metabolomics could yield findings closer to the actual cellular situation. Therefore, metabolites are rapidly emerging as the valuable biomarkers for early PCa screening [18].

Considering these perspectives, we collected plasma from tumor-free controls (TFCs), PCa patients, and CRPC patients and isolated exosomes from the collected samples. Subsequently, we used a combination of proteomic and metabolomic techniques to analyze the exosome expression profiles in these groups. The aim of this study was to identify biomarkers that can distinguish disease classifications at the protein and metabolic levels and to further explore their value for PCa precise diagnosis. Moreover, combined multi-omics analysis was used to further understand the molecular profiles of PCa at different stages. Finally, the functional role of exosomal protein LRG1 was studied in prostate cancer.

## Materials and methods

### Clinical samples

All participants were recruited from Shenzhen Hospital of Southern Medical University (Shenzhen, China), Hainan General Hospital (Hainan China), Shenzhen’s People Hospital (Shenzhen, China), and The Second Hospital of Tianjin Medical University (Tianjin, China). The study was approved by the human ethics committees of these hospitals. Written informed consent and clinical information were obtained from all patients. Serum samples (3–4 mL) were obtained from TFCs and PCa and CRPC patients and stored at − 80 °C until processing. TFCs were age-matched normal individuals or men whose prostates were free of cancer (e.g., individuals with benign prostatic hyperplasia [BPH]).

### Exosome isolation and identification

Exosomes derived from patient serum were obtained using the classical ultracentrifugation approach [19]. First, living and dead cells were removed by low-speed centrifugation (300 × *g* for 10 min and 2000 × *g* for 10 min successively) at − 4 °C. Next, the cell debris was centrifuged and removed at 10,000 × *g* for 30 min. Then, the obtained supernatant was filtered by a 0.22-µm filter to further purify exosomes. The rest of the supernatant was ultracentrifuged at 100,000 × *g*/70 min with a TI70 rotor. Second, the supernatant was discarded again, purified exosomes were resuspended in PBS, and exosome pellets were obtained following ultracentrifugation at 100,000 × *g*/70 min with a TI70 rotor. After the supernatant was discarded, an appropriate amount of PBS was added according to the initial plasma volume to resuspend exosomes, and the protein concentration was measured after packaging and stored at − 80 °C.

### Trypsin digestion

On the basis of the protein concentration, 8 M urea was added to equal amounts of total protein, and then adjusted to the same volume. Next, DTT was added to the protein solution to a final concentration of 5 mM, and the solution was incubated at 56 °C for 30 min. After cooling to room temperature, IAM was added to a final concentration of 11 mM, and the solution was incubated at room temperature for 15 min in the dark. The protein sample was then diluted by adding 100 mM TEAB to a urea concentration of less than 2 M. Finally, trypsin was added at a 1:50 trypsin-to-protein mass ratio for the first digestion overnight and a 1:100 trypsin-to-protein mass ratio for a second 4 h digestion.

### Liquid chromatography–tandem mass spectroscopy analysis for proteomics

The tryptic peptides were dissolved in 0.1% formic acid (solvent A) and directly loaded onto a home-made reversed-phase analytical column (length, 15 cm; internal diameter, 75 µm). The gradient consisted of an increase from 6 to 23% solvent B (0.1% formic acid in 98% acetonitrile) over 26 min, from 23 to 35% over 8 min, from 35 to 80% over 3 min, and then holding the concentration at 80% for the last 3 min, all at a constant flow rate of 400 nL/min on an EASY-nLC 1000 ultra-performance liquid chromatography (UPLC) system. The peptides were subjected to a nanospray ionization (NSI) source followed by tandem mass spectrometry (MS/MS) in Q ExactiveTM Plus (Thermo) coupled online to the UPLC. The electrospray voltage applied was 2.0 kV. The m/z scan range was 350 to 1800 for the full scan, and intact peptides were detected in the Orbitrap at a resolution of 70,000. Peptides were then selected for MS/MS by using the normalized collision energy (NCE) setting as 28, and the fragments were detected in the Orbitrap at a resolution of 17,500. A data-dependent procedure was performed that alternated between one MS scan followed by 20 MS/MS scans with a 15.0-s dynamic exclusion. Automatic gain control (AGC) was set at 5E4. The fixed first mass was set to 100 m/z.

### Extraction of metabolites

The resuspended exosomes in PBS were added to 1000 μL of extract solution (acetonitrile:methanol:water = 2:2:1). After repeated freezing and thawing three times with liquid nitrogen, all samples were vortexed for 30 s before sonication for 10 min. After standing for one hour at 40 °C, the samples were centrifuged at 12,000 rpm at 4 °C for 15 min, after which 950 μL of supernatant was dried in a vacuum concentrator, and an extract solution (methanol:acetonitrile:water = 2:2:1) containing an isotopically labeled internal standard mixture was added in proportion. After 30 s of vortexing and 10 min of sonication, the obtained samples were centrifuged at 12,000 rpm at 4 °C for 15 min. Subsequently, the supernatant was obtained and transferred to a fresh glass vial for liquid chromatography–mass spectroscopy (LC–MS) analysis. The quality control (QC) sample was prepared by mixing an equal aliquot of the supernatant from all samples.

### LC–MS/MS analysis for metabolomics

LC–MS/MS analysis was performed using a UHPLC system (Vanquish, Thermo Fisher Scientific) with a UPLC BEH Amide column (2.1 mm × 100 mm, 1.7 μm) coupled to a Q Exactive HFX mass spectrometer (Orbitrap MS, Thermo). The mobile phase consisted of 25 mmol/L ammonium acetate and 25 mmol/L ammonia hydroxide in water (pH = 9.75) (A) and acetonitrile (B). The analysis was conducted with the following elution gradient: 0 ~ 0.5 min, 95% B; 0.5 –7.0 min, 95% ~ 65% B; 7.0 –8.0 min, 65% ~ 40% B; 8.0 – 9.0 min, 40% B; 9.0 –9.1 min, 40% ~ 95% B; 9.1 –12.0 min, 95% B. The column temperature was maintained at 30 °C. The auto-sampler temperature was 4 °C, and the injection volume was 2 µL. The QE HFX mass spectrometer was used for its ability to acquire MS/MS spectra in the information-dependent acquisition (IDA) mode under the control of acquisition software (Xcalibur, Thermo). In this mode, the acquisition software continuously evaluated the full-scan MS spectrum. The ESI source conditions were set as follows: sheath gas flow rate, 50 Arb; Aux gas flow rate, 10 Arb; capillary temperature, 320 °C; full MS resolution, 60,000; MS/MS resolution, 7500; collision energy, 10/30/60 in NCE mode; spray voltage, 3.5 kV (positive) or − 3.2 kV (negative).

### Western blotting and semi-quantification

The detailed steps of western blotting have been described previously. Briefly, the cell pellet was collected in RIPA buffer with 0.1 M DTT. After 30 min of incubation in an ice-water bath, 5×loading buffer was added to the protein solution and heated for 5 min at 95 °C. To maintain the consistency of the loading control, 5 µg of total protein from resuspended exosomes was loaded for each experiment. For cell lines, BPH1, LNCaP, and LNCaPAI cell lysates were prepared using the same protocol. After centrifugation for 5 min at 12,000 × *g*, the obtained protein supernatant was subjected to SDS-PAGE, and the gel was then blotted onto a PVDF membrane. Membranes were blocked with 5% skim milk for 1 h at room temperature. The primary antibodies against CD9, TSG101, calnexin, and GAPDH were separately incubated with the membrane at 4 °C overnight. On the next day, HRP-conjugated secondary antibody (1:5000 anti-rabbit or 1:10,000 anti-mouse dilution) incubation was performed for 1 h, and the intensity was measured using an imaging system (Bio-Rad ChemiDocTM Imaging System).

### Receiver operating characteristic curve

A receiver operating characteristic (ROC) curve was used to further assess the potential diagnostic value of proteins and metabolites derived from patients’ plasma. A combined protein and metabolite panel was constructed to improve the performance of the disease classifier.

### Cell culture and lentivirus packaging

PC-3 and 293 T were grown in DMEM containing 10% FBS and 1% penicillin/streptomycin. DU145 and LNCaP were cultured in RPMI1640 medium with 10% FBS and 1% penicillin/streptomycin. Primary HUVEC cells were cultured in endothelial cell medium (ECM, Cat. #1001, ScienCell Research Laboratories, Inc). All the cells were maintained at 37 °C with 5% CO_2_. All the steps of lentivirus packaging were according to the previously described. LRG1 cDNA was obtained using Transcription High Fidelity cDNA Synthesis Kit and then subcloned into PCDH-CMV-Puro-EGFP vector.

### IHC staining

Immunohistochemical (IHC) analysis was carried out to assess PCa tumor tissues and adjacent para-cancerous tissues. Briefly, the tissue microarray was put into a repair box filled with citric acid antigen repair buffer (pH 9.0) for antigen repair, and then it was cooled down to room temperature. LRG1 primary antibody were incubated at 4 °C overnight (Cat. #ab178698). The next day, secondary HRP-conjugated antibody were incubated for 1 h. Finally, counterstaining was performed with haematoxylin. The criteria of tissues scoring was performed as previously described.

### Gene expression analysis by reverse transcription-quantitative PCR (RT-qPCR)

Total RNA was isolated with EasyPure RNA Kit (Transgene, Cat. #ER101). Immediately after, RNA reverse transcription was carried out using PrimeScript™ RT (TaKaRa, Cat. #RR047A). PCR amplification was performed using SYBR green fluorescent dye (Transgene, Cat. #AQ142-21). Primer sequences are provided as follow:

LRG1: F: 5'-GTTGGAGACCTTGCCACCT-3', R: 5'-GCTTGTTGCCGTTCAGGA-3';

GAPDH: F: 5'-ACAACTTTGGTATCGTGGAAGG-3', R: 5'-GCCATCACGCCACAGTTTC-3'.

### Cell proliferation and colony formation assay

Cells were seed into 96-well plates (5000 cells/well). Cell viability was measured by CCK8 reagent (FC101-01, TransGen Biotech Co., Ltd) at various time points (0 h, 24 h, 48 h, and 72 h). 10 μl CCK8 was added into culture medium. After 2 h incubation, the absorbance of each well was measured at 450 nm. For colony formation assay, cells were digested into single cells by 0.25 trypsin, which were further counted by a hemacytometer. Cells (1000 cells/well) in log growth phase were plated into 6 well plate. After 14 days, cells were washed, fixed in 4% paraformaldehyde for 30 min, and then stained with crystal violet solution.

### Wound healing and transwell assay

Cells were plated into 6-well plate. When the cells grew to 100% confluence, a sterilized tip was used to gently draw lines on the plate. After 0 h, 24 h and 48 h, the wound healing area was calculated using Image-Pro PlusV1.8.0. For transwell assay, cells were cultured in RPMI 1640 medium without FBS. After 24 h, cells (2 × 10^5^ LNCaP; 2 × 10^4^ PC3) were seed into a 24-well plate. The upper chamber membranes (labselect, 8.0 μm pores #14342) were coated with matrigel (BD, #356230) and the lower chamber was supplemented with FBS. After a period of culture, cells on the bottom side of the chamber membrane were fixed, stained with crystal violet and photographed. Relative invasion ability was calculated as cell number per five random visual fields.

### Tube formation assay

We performed pretreatment for removal of the exosomes derived from commercial fetal bovine serum. In brief, the exosomes of fetal bovine serum were eliminated by ultracentrifugation, then collecting the supernatant for cell culture. Subsequently, PCa cells were cultured in DMEM 10% FBS medium without exosomes. After 48 h, the exosomes derived from PCa cell lines were enriched by ultracentrifugation and applied for further angiogenesis experiment. HUVEC cells were cultured in RPMI1640 medium without FBS. Meanwhile, exosomes (20 μg/ml) derived from different cell lines were added to culture medium. After 24 h cell culture, HUVEC cells were harvested and counted, then 1 × 10^4^ HUVEC cells were plated into Matrigel (BioCoat, #356231) coated 96-well plate. After 4 h and 8 h incubation, the number of branch points were measured to quantify tube formation.

### Statistical analysis

Multiple group comparisons were performed using one-way ANOVA followed by Tukey–Kramer tests. P < 0.05, shown in the figures as “*”, was considered to indicate statistical significance. Consistently, P < 0.01 (**), P < 0.001 (***), and P < 0.0001 (****) indicated highly significant findings. Statistical analyses were performed using the GraphPad Prism 7.04.

## Results

### Clinical characteristics of patients and validation of exosomes

**Fig. 1:**
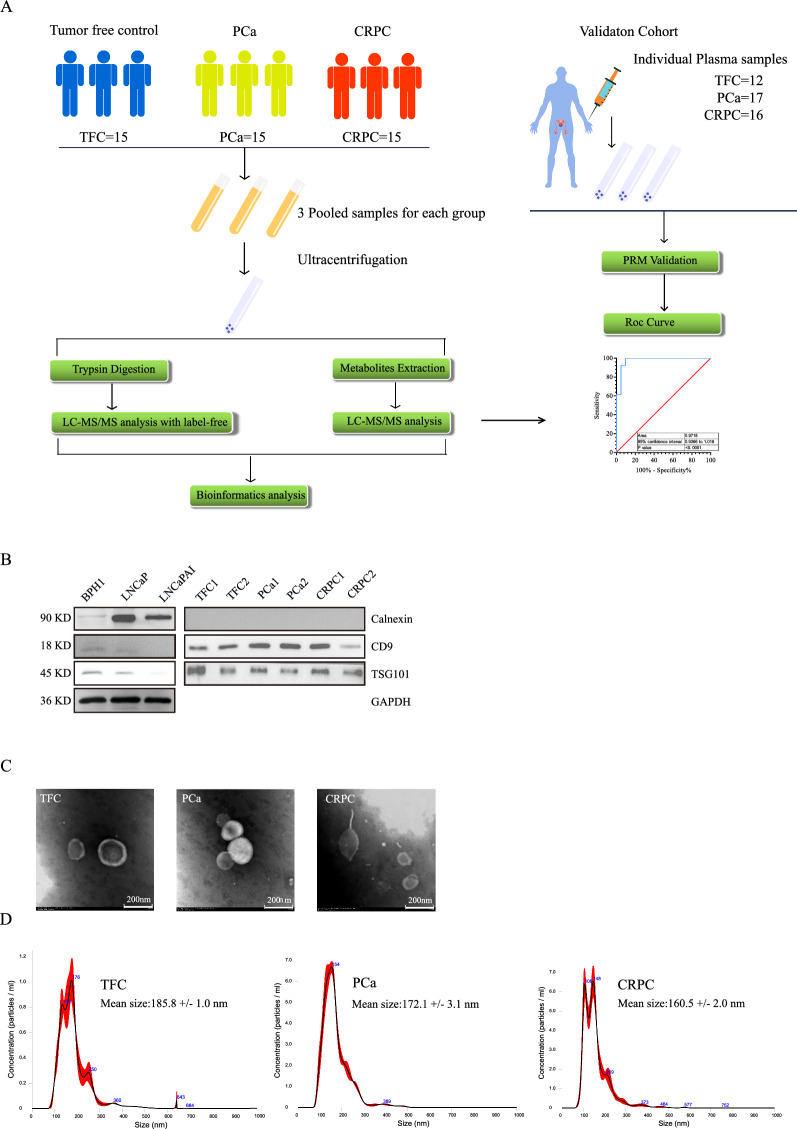
Flow Chart of exosome isolation and identification. **A** Flow Chart of exosome isolation and omics analysis strategy. **B** Exosomal markers TSG101, CD9 and Calnexin were detected by Immunoblot. **C** Morphology observation of exosomes by Transmission Electron Microscopy. **D** Exosome concentration and size were measured by the Nanoparticle Tracking Analysis system

In our study, a total of 90 plasma samples, including 45 (15 TCF, 15 PCa, and 15 CRPC) in the discovery set and 45 (12 TCF, 17 PCa, and 16 CRPC) in the validation set, were recruited to identify exosomal protein markers, of which 52 samples (18 TFC, 21 PCa, and 13 CRPC) were recruited to identify exosomal metabolic biomarkers (Fig. 1A). For the exosome protein biomarker screening study, 4–5 plasma samples were mixed into one pooled sample according to clinical treatment stage to avoid individual differences. Thus, nine pooled samples in total (TCF = 3, PCa = 3, CRPC = 3, the volume of each sample was approximately 12–15 mL) were used for exosome isolation via ultracentrifugation and subjected to the LC–MS/MS proteomics study. For the exosomal protein validation and metabolic screening studies, the exosomes from individuals were extracted separately. Exosome extraction was performed as previously described [20]. The basic clinical characteristics of the patients, including age, PSA level, Gleason score, and local treatment, were listed in Table 1.

**Table 1:** Patient and tumour characteristics for trainning cohort

	TFC (n = 15)	PCa (n = 15)	CRPC (n = 15)
Age (years)			
Median (range)	54 (50–75)	73.5 (52–87)	75 (66–82)
Baseline PSA (ng/ml)			
Median (range)		8.6 (0.004–167)	23.04 (0.36–100)
Local treatment, n (%)			
Endocrinotherapy		10 (67%)	11 (73%)

The basic characteristics of resuspended exosomes were identified by transmission electron microscopy (TEM), nanoparticle tracking analysis (NTA), and western blotting (WB). As shown in Fig. 1C, plasma-derived exosomes were clearly observed in each sample. The particle size was approximately 50–150 nm, and partial exosomes presented with a classic saucer shape [21]. In addition, NTA results demonstrated that after a 0.22-µm filtration, all the samples exhibited good homogeneity, and most of the particles were distributed in the particle size range of 50–150 nm. The size of a few particles was approximately 200–400 nm, but the percentage of such particles was relatively low (Fig. 1D). To further validate our exosomal preparation, CD9, TSG101, and calnexin markers were detected by WB to characterize patient-derived exosomes. As shown in Fig. 1B, TSG101 and CD9 were strongly detected in patient-derived exosome samples, but relatively weak bands were observed in the cell lines. However, for the exosomal negative marker calnexin, strong bands were detected in cell line lysate samples, in contrast to those observed in the exosomes of patient plasma. These results indicated that the patient-derived exosomes were of good quality and purity, and they were suitable for subsequent proteomic and metabolomic analyses.

### Exosomal proteins are dysregulated in aggressive prostate cancer

**Fig. 2:**
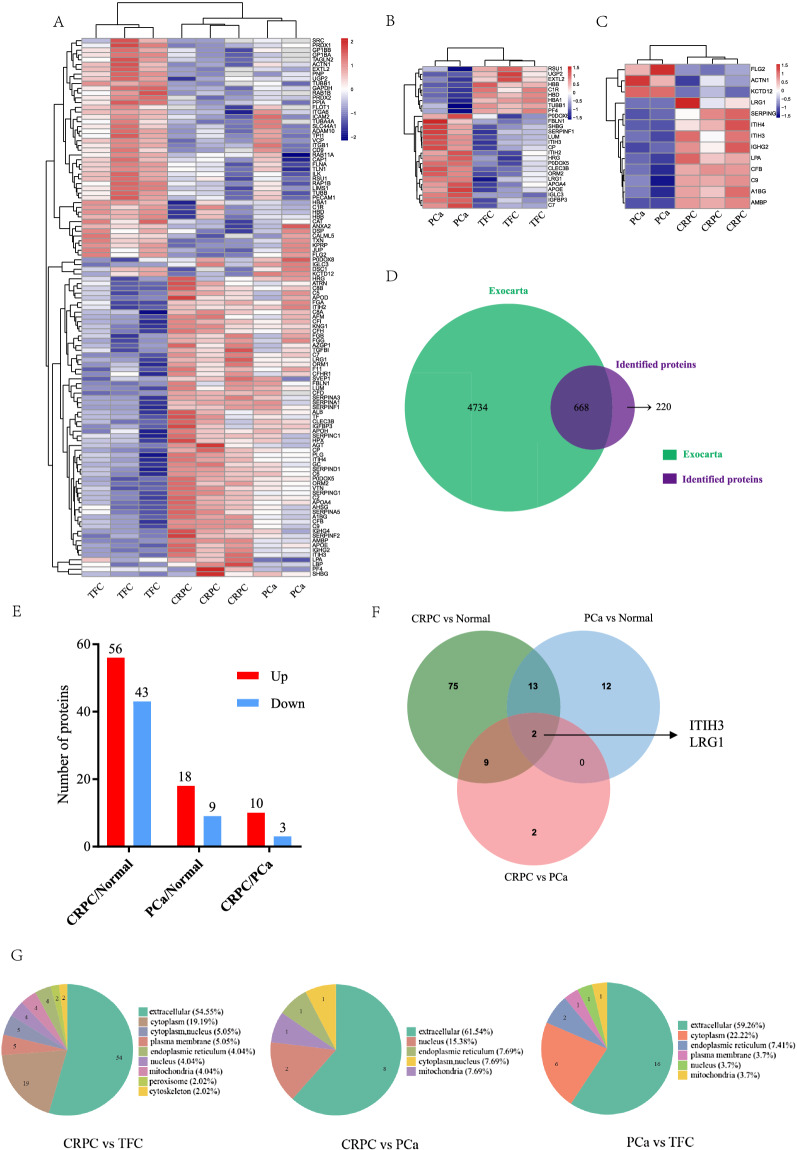
Overview of Hierarchical Clustering analysis based on differential protein expression derived from plasma exosomes. **A**–**C** The respective heatmaps were constructed according to relative abundance ratios. **D** The overlapping proteins between Exocarta database and the proteins identified by proteomic-based MS technolog. **E** Differential protein expression statistics of proteomic data demonstrated significantly differentially expressed proteins (log_2_ (fold change) > 1.5 or log_2_ (fold change) <  − 1.5 and p-value < 0.05). **F** Venn diagram of the number of differentially expressed proteins, LRG1and ITIH3. **G** Subcellular localization of differentially expressed proteins from plasma exosomes, CRPC/TFC, CRPC/PCa and PCa/TFC

Differential exosomal proteins (DEPs) between the different groups were identified by LC–MS/MS analysis in the discovery cohort. A significant difference was defined as log_2_ (fold change) > 1.5 or log_2_ (fold change) <  − 1.5 and p-value < 0.05. Among the three pooled PCa subgroups, PCa3 patients were metastatic and excluded for significance analysis as their data deviated from the main cohort. The heatmaps generated on the basis of the DEPs are shown in Fig. 2. A total of 938 proteins were identified from plasma exosomes, out of which 668 proteins overlapped with ExoCarta database (Fig. 2D). As is shown in heatmap, in the comparison between TFCs and PCa patients, 18 upregulated and 9 downregulated DEPs were found in PCa patient-derived purified exosomes (Fig. 2B). Meanwhile, compared to TFCs, 56 DEPs were upregulated and 43 DEPs were downregulated in CRPC patients, (Fig. 2A). However, fewer DEPs were observed in the comparison between CRPC and PCa patients, which showed 10 remarkably upregulated and 3 downregulated proteinsin the CRPC group (Fig. 2C and E). The Venn diagram shows the number and overlap of proteins in the various comparisons (Fig. 2F). More than half of the proteins were predicted to be localized in the extracellular region (Fig. 2G).

To better understand the protein-level changes in each group, Gene Ontology (GO) enrichment analysis was performed to determine the biological significance of these abnormally expressed proteins. As shown in Additional file 1: Fig. S1, in the comparison of PCa and TFC, nucleotide sugar metabolic process, oxygen transport, and gas transport were significantly enriched. Additionally, in the comparison between CRPC and PCa, the differentially expressed proteins were mainly related to mucopolysaccharide metabolic processes, glycosaminoglycan metabolic processes, as well as regulation of the humoral immune response.

### Potential diagnostic value of the combination of exosomal proteins LRG1 and ITIH3 for CRPC

**Fig. 3:**
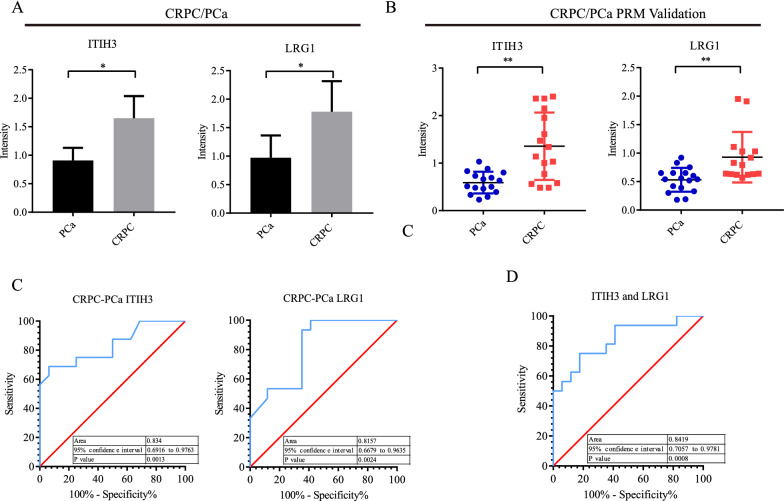
Bar graphs summarize the quantification of LRG1 and ITIH3 levels in each group and ROC curve analysis for individual proteins and combined proteins panel. **A** Untargeted proteomics exhibited the relative quantification of LRG1 and ITIH3. **B** PRM Validation of LRG1 and ITIH3 by independent cohort TFC = 21, PCa = 15 and CRPC = 12. **C** ROC curve showed the overall performance of the classifier of ITIH3 and LRG1 respectively. **D** ROC curve of ITIH3 combined with LRG1

To confirm the results of untargeted proteomics analyses, plasma samples from 45 patients (TFC: n = 12; PCa: n = 17; and CRPC: n = 16) were collected for parallel reaction monitoring (PRM) validation. Patient information is listed in Table 2. A total of 21 potential protein candidates were validated by PRM-targeted analysis. The results showed that for the comparison of PCa patients and TFCs, the trends of the five differential proteins were similar to the untargeted proteomics results. The expression levels of these proteins did not show significant differences in PRM validation. In addition, for the comparison between CRPC and PCa, similar trends were observed for 15 validated proteins from CRPC samples. Interestingly, LRG1 and ITIH3 protein expression levels in the CRPC group were 1.5- and 2.04-fold higher than those in the PCa group (Fig. 3A, B).

**Table 2:** Patient and tumour characteristics for proteomics validation

	TFC (n = 12)	PCa (n = 17)	CRPC (n = 15)
Age (years)			
Median (range)	66.5 (46–77)	70.5 (53–83)	71 (55–83)
Baseline PSA (ng/mL)			
Median (range)		10.08 (0.13–149)	120 (5.9–137.9)
Local treatment, n (%)			
Radical prostatectomy		15 (88.2%)	
Endocrinotherapy			8 (53.33%)

To study the potential clinical value of DEPs, ROC curve analysis was performed to identify whether LRG1 and ITIH3 could be used as biomarkers for disease diagnosis and classification. Results demonstrated that the AUC values of LRG1 and ITIH3 were 0.834 and 0.815, respectively, for distinguishing CRPC from PCa (Fig. 3C). To explore a more effective biomarker model for diagnosis, biomarker panels were constructed to show superior performance by binary logistic regression analysis. Interestingly, the combination of LRG1 and ITIH3 obtained an AUC of 0.842 (Fig. 3D), suggesting that the combination of these two exosomal proteins could be a potential protein marker panel to distinguish CRPC from PCa.

### Abnormal metabolites were identified in the exosomes derived from PCa and CRPC patients

Metabolic abnormalities are closely associated with disease processes. Arguably, metabolite changes more directly reflected the state of various periods of PCa and CRPC [22]. In the present study, we were interested in studying the differentially enriched metabolic profiles of exosomes in PCa and CRPC. Information regarding the patients evaluated in these analyses is provided in Table 3. The LC–MS/MS metabolomics analysis demonstrated that a total of 206 substances were matched with the secondary mass spectrometry (MS2) database, and 86 substances and 120 substances were detected in positive-ion mode (POS) and negative-ion mode (NEG) analyses, respectively. Specifically, there were 89 lipids and lipid-like molecules (43%), 28 organic acids and derivatives (13.5%), 20 organoheterocyclic compounds (9.7%), 20 benzenoids (9.7%), and nine organic oxygen compounds (4.3%).

**Table 3:** Patient and tumour characteristics of metabolomics

	TFC (n = 18)	PCa (n = 21)	CRPC (n = 13)
Age(years)			
Median (range)	56 (37–80)	74 (58–82)	74 (46–88)
Baseline PSA (ng/mL)			
Median (range)	3.389 (0.41–14.7)	33.64 (6.66–284)	83.33 (22.86–101)
Post-treatment PSA (ng/ml)			
Median (range)			0.955 (0.01–37.33)
Gleason score, n (%)			
≤ 6		4 (19.05%)	1 (7.69%)
7		7 (33.33%)	1 (7.69%)
≥ 8		3 (14.29%)	3 (23.08%)
Local treatment, n (%)			
Radical Prostatectomy		6 (28.57%)	
Endocrinotherapy		9 (42.86%)	

**Fig. 4:**
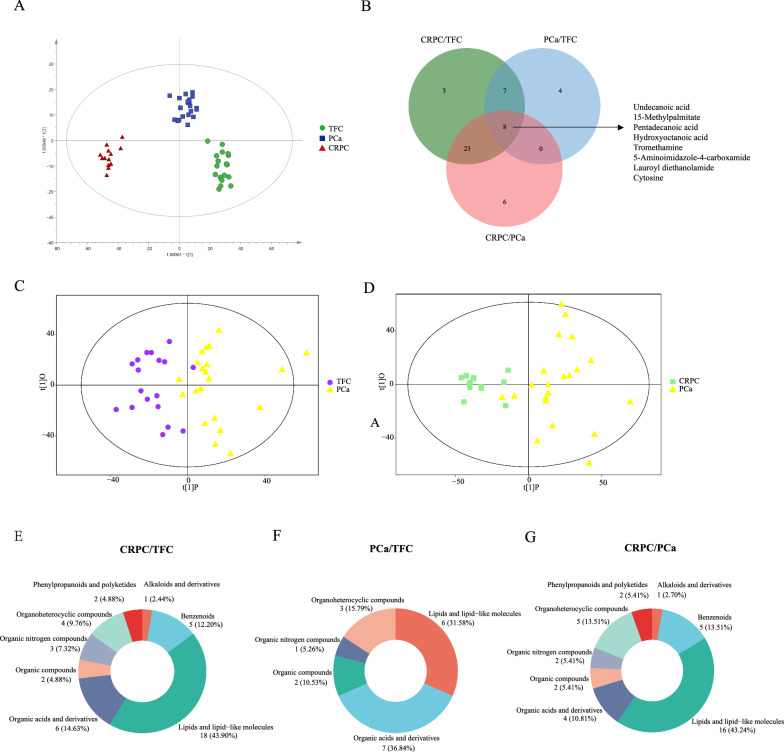
An orthogonal partial least-squares discriminant analysis (OPLS-DA) model of plasma exosome metabolites. **A** Overview of OPLS-DA, including three independent groups TFC, PCa, and CRPC. **B** Venn diagram display of overlapping differentially expressed metabolites. **C** OPLS-DA for CRPC/PCa. **D** OPLS-DA for PCa/TFC. **E**–**G** The pie chart represents the proportion of each separate class of metabolites, CRPC/TFC, CRPC/PCa, and PCa/TFC

**Fig. 5:**
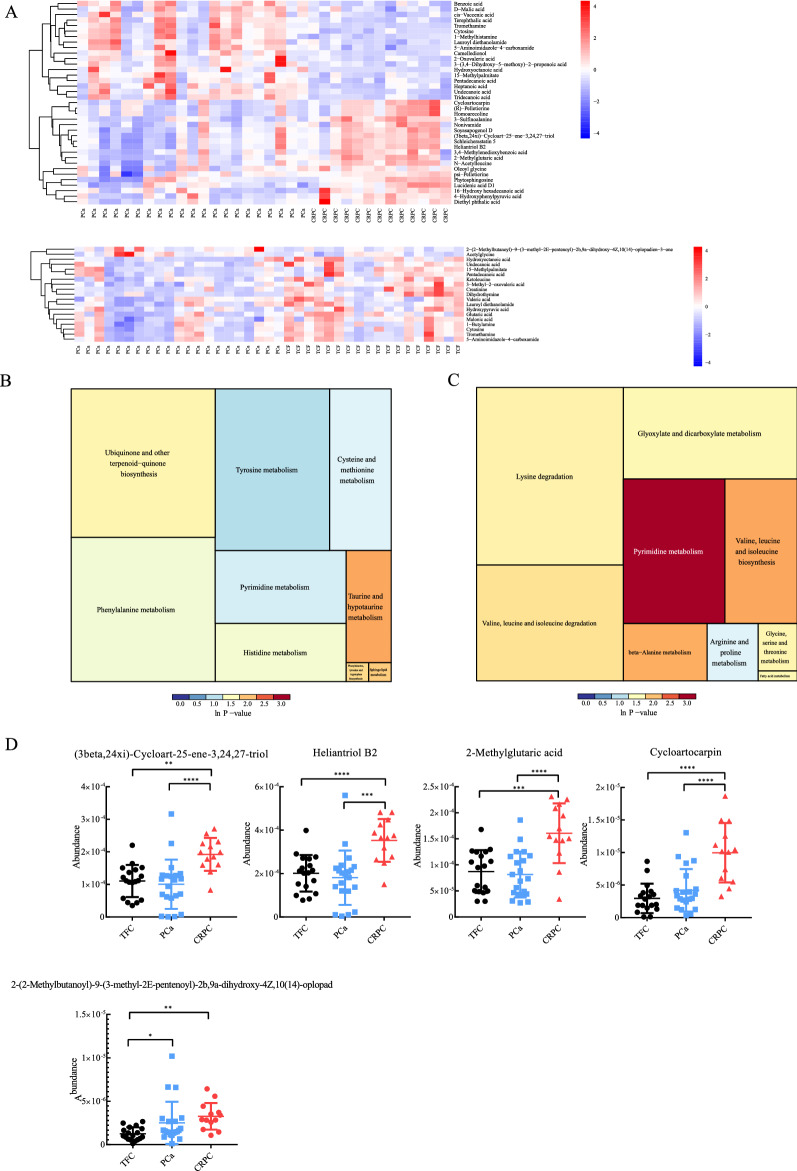
Differential expressed metabolites were displayed in the heatmap and performed for signaling pathways enrichment analysis. **A**, **B** the heatmap was constructed according to the date of CRPC/PCa and PCa/Normal. **C** Signaling pathways enrichment analysis for each comparison

A total of 97 differential exosomal metabolites (DEMs) were identified among the three groups. Based on the DEMs, orthogonal projections to latent structures-discriminant analysis (OPLS-DA) were performed to draw a score scatter plot. The overview of OPLS-DA plots based on DEMs revealed clear segregation between comparable groups (Fig. 4A). In addition, an OPLS-DA model was constructed to summarize the clear clustering in the comparison of PCa patients and TFCs (Fig. 4C) and patients with CRPC and those with PCa (Fig. 4D). The Venn diagram shows the number and overlap of exosomal metabolites in the various comparisons (Fig. 4B). The pie charts represented the percentage of metabolites in each comparable group (Fig. 4E–G). DEMs were defined by a fold change > 1.3 or < 0.7 in relative abundance and a p-value < 0.05. A heatmap of the top DEMs revealed that 19 substances with significant changes were identified in the PCa versus TFC cohort (Fig. 5A, bottom panel). The metabolites in the PCa samples showed a decreasing trend in most cases, and only 2-(2-Methylbutanoyl)-9-(3-methyl-2E-pentenoyl)-2b,9a-dihydroxy-4Z,10(14)-oplopadien-3-one and acetylglycine showed levels 2.01- and 1.48-fold higher than those in the TFC group. The dihydrothymine, creatinine, and hydroxyoctanoic acid contents in the PCa group were significantly (0.39-, 0.42- and 0.48-fold, respectively) lower than those in the TFC group.

For the comparison of CRPC versus PCa, the heatmap plot showed 37 metabolites with significant differences (Fig. 5B, upper panel, 19 upregulated and 18 downregulated). Among the 19 metabolites accumulated in the CRPC group, 12 of which were higher more than 1.5-fold in comparison with the levels in PCa samples. In addition, cycloartocarpin showed significant changes in the CRPC groups, and its accumulation in exosomes was 2.37-fold higher than that in the PCa group. Among the reduced exosomal metabolites, the levels of tridecanoic acid, undecanoic acid, and hydroxyoctanoic acid decreased to nearly half in the CRPC samples (0.51, 0.49, and 0.34, respectively) (Fig. 5D).

### Potential diagnostic value of differential metabolites for PCa and CRPC

**Fig. 6:**
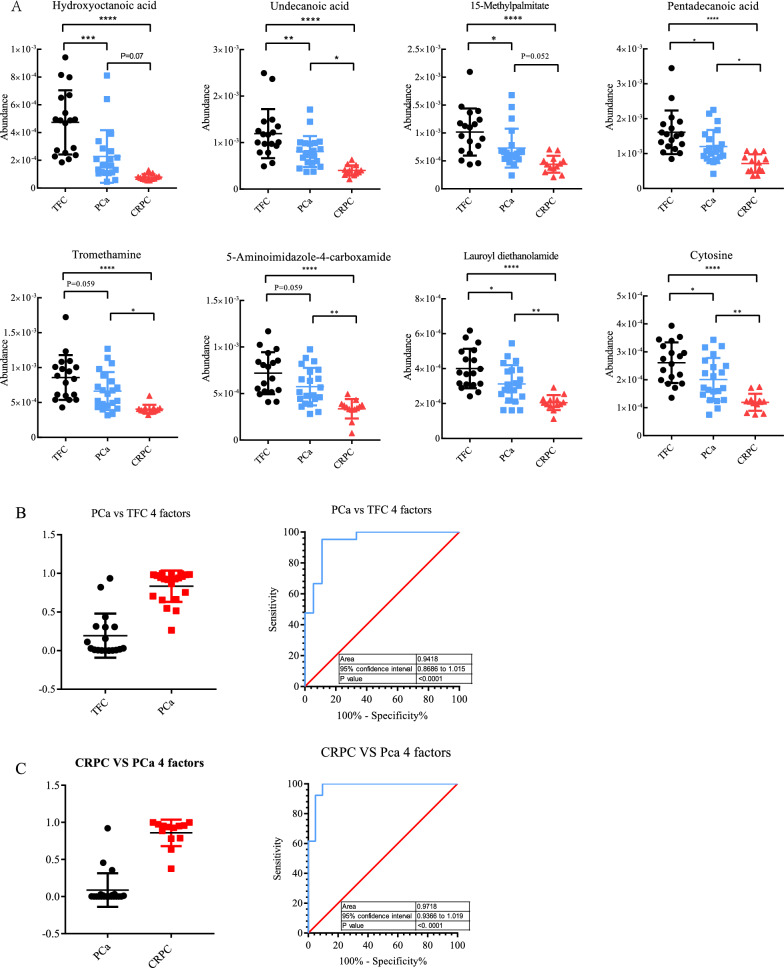
Differentially-expressed metabolites by Column scatter plots and ROC curve analysis for individual metabolites and combined metabolites panel. **A** The level of metabolites was observed to significantly changed in CRPC and  groups compared with TCF group.  **B, C** Overall performance of the classifier of combined metabolites panel

ROC curve analysis was performed to further characterize the predictive value of these differential metabolites. First, in comparisons of PCa versus TFC, metabolites showing fold-change values > 1.3 or < 0.7 were included as potential candidates. The results showed three metabolites with AUC values > 0.7 and < 0.8, namely, 2-(2-methylbutanoyl)-9-(3-methyl-2E-pentenoyl), acetylglycine, and undecanoic acid. Additionally, dihydrothymine, creatinine, hydroxyoctanoic acid, and 3-methyl-2-oxovaleric acid had a relatively high AUC values > 0.8. In contrast, for the comparison of CRPC and PCa, a large number of differential metabolites were identified. Therefore, the standard for identifying differential metabolites was more strict (fold-change values > 1.5 or < 0.6). Consequently, the AUC values of nine metabolites was still higher than 0.8, of which seven metabolites (cycloartocarpin, 2-methylglutaric acid, heliantriol B2, (3beta,24xi)-Cycloart-25-ene-3,24,27-triol, hydroxyoctanoic acid, 1-methylhistamine, 5-aminoimidazole-4-carboxamide) with AUC values greater than 0.85. Additionally, heliantriol B2 and hydroxyoctanoic acid showed AUC values of 0.886 (Fig. 6A).

In the preset study, we also tried to distinguish CRPC from PCa by using a multiple-metabolite combination model and compare their sensitivity and specificity. For PCa and CRPC cohort, four metabolite combinations were selected for the logistic regression analysis. The results showed that for PCa diagnosis, the AUC value reached 0.9418 (Fig. 6B), which was higher than the accuracy of a single metabolite for PCa diagnosis. For CRPC diagnosis, the prediction model based on the combination of the four metabolites achieved an higher value of 0.978 (Fig. 6C), which was theoretically far more accurate than PSA-based screening. Therefore, these combinations of metabolites may better differentiate the progression of PCa and CRPC.

### Differentially expressed exosomal proteins and metabolites were correlated in CRPC

**Fig. 7:**
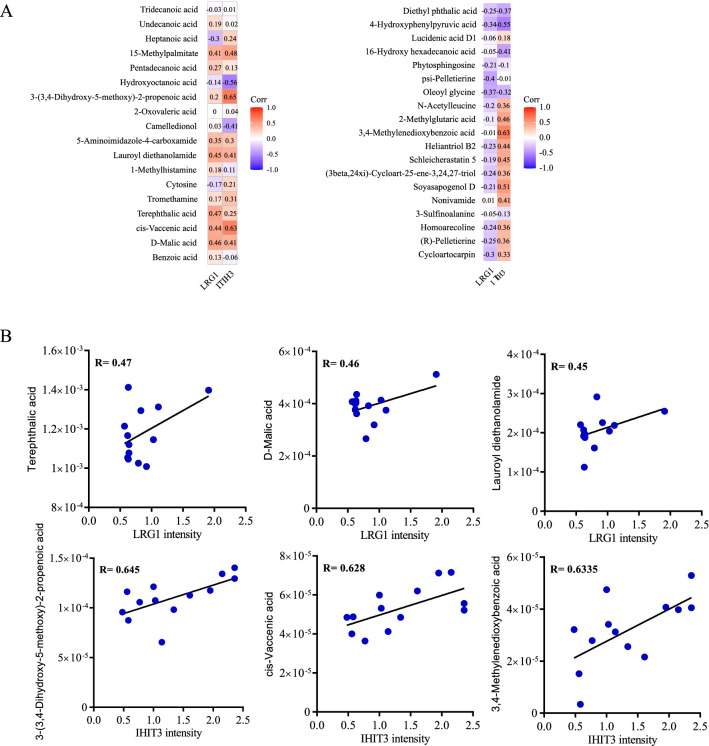
Heatmap and correlation analysis presenting the association between the proteins and the metabolites

In the present study, we also attempted to analyze the associations between the differentially expressed exosomal proteins and metabolites, since the functional variation of these two molecules may affect each other in disease progression. The heatmap graph of the correlation analysis between proteins and metabolites is provided in Fig. 7. First, the correlations between APOE and the related metabolites such as 4-hydroxyphenylpyruvic acid (R = 0.43, P = 0.08), lucidenic acid D1 (R = 0.32, P = 0.19), and benzoic acid (R = − 0.34, P = 0.18) were analyzed. However, only 4-hydroxyphenylpyruvic acid’s p-value is closed to 0.05 (Additional file 1: Fig. S2). For analyses involving LRG1, terephthalic acid (R = 0.47, P = 0.022), D-malic acid (R = 0.46, P = 0.001), and lauroyl diethanolamide (R = 0.45, P = 0.001) showed positive correlations with LRG1 expression. For analyses with ITIH3, 3-(3,4-dihydroxy-5-methoxy)-2-propenoic acid (R = 0.645, P = 0.022), cis-vaccenic acid (R = 0.628, P = 0.001), and 3,4-methylenedioxybenzoic acid (R = 0.6335, P = 0.001) exhibited relatively high R-values, suggesting a potential link between the differential proteins and metabolites in prostate cancer exosomes (Fig. 7A, B).

### LRG1 shows upregulation in advanced prostate cancer

**Fig. 8:**
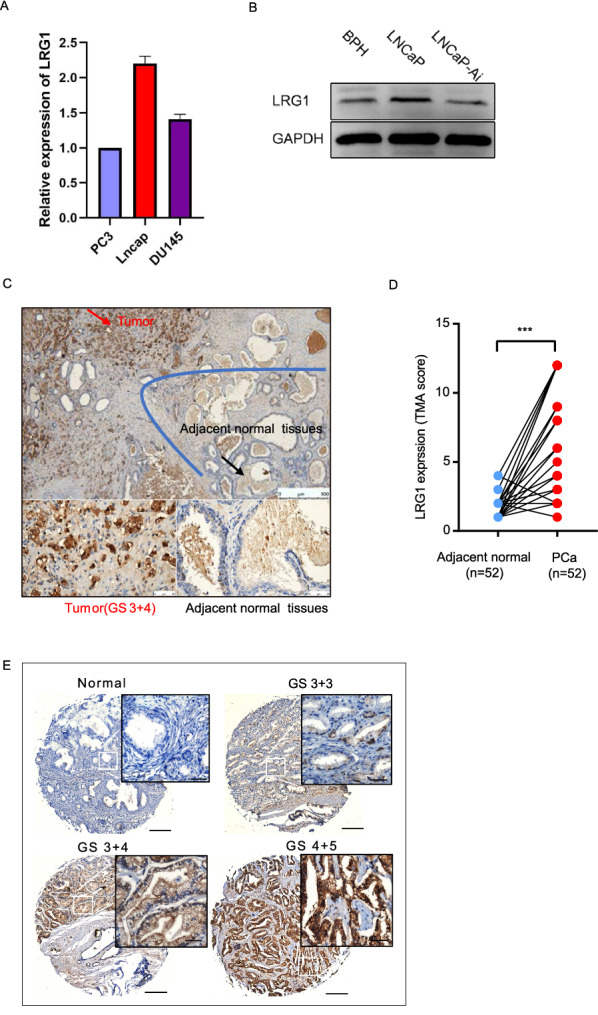
LRG1 shows upregulation in advanced prostate cancer. **A**, **B** The LRG1 expression levels in PCa cell lines were measured by RT-PCR and Western Blot. **C** LRG1 positive staining was mainly localized to cytoplasm and membrane of PCa cells. **D** The expression level of LRG1 in 52 paired PCa tissue microarray (TMA). **E** The expression level of LRG1 in different Gleason score groups

Considering the importance of exosomes as messenger carrier in tumor development and the exosomal protein LRG1 in diseases including cancer [23], we investigated the function of LRG1 in PCa progression. We first analyzed the expression level of LRG1 in cell lines and PCa clinical tissues. Results showed that LNCaP exhibited relatively high LRG1 expression level (Fig. 8A, B). Cellular distribution and expression level of LRG1 was studied in PCa tissue microarray (TMA) by immunohistochemical staining, which contained paraffin embedded 52 PCa samples with paired adjacent non-tumor samples, 42 unpaired PCa samples and 3 normal prostate samples. The result showed that LRG1 positive staining was mainly detected in cytoplasm and membrane of PCa cells (Fig. 8C). Additionally, the expression level of LRG1 in PCa tissues were significantly higher than paired adjacent normal tissues(Fig. 8D, P < 0.0001). Moreover, LRG1 expression was positively correlated with Gleason score (Fig. 8E, P < 0.0001).

### LRG1 expression promotes malignant phenotypes in PCa cells

**Fig. 9:**
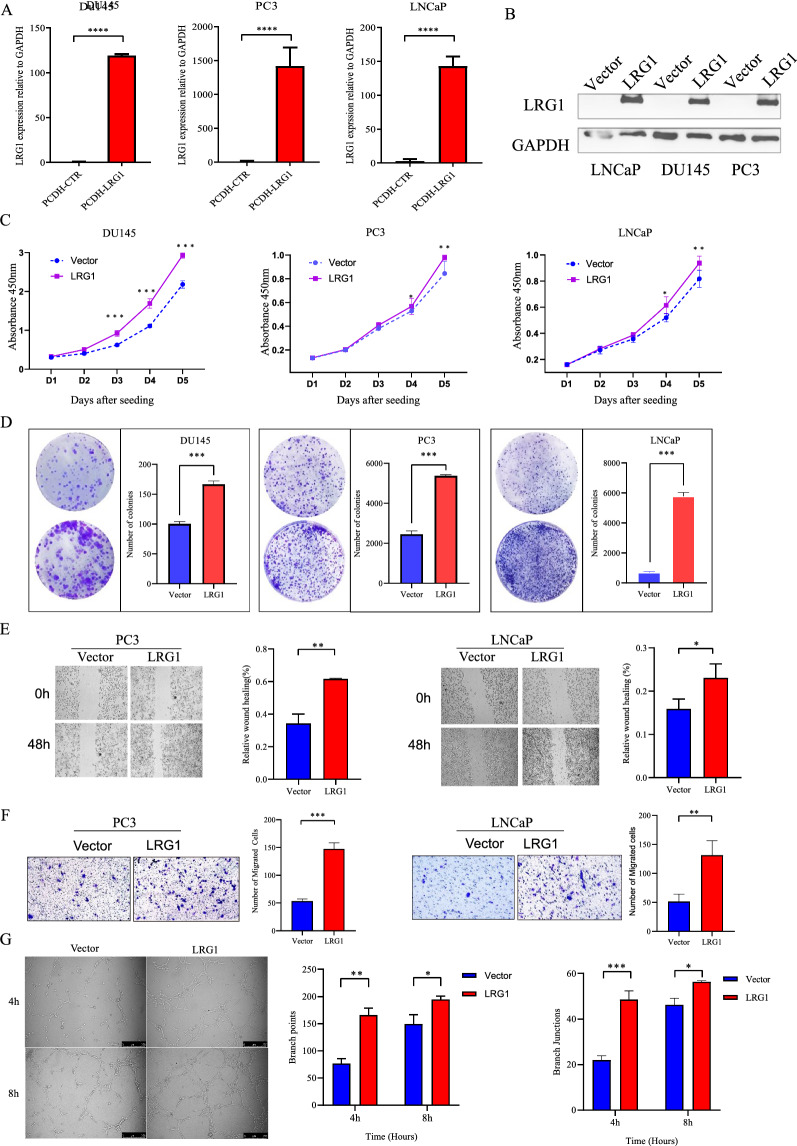
LRG1 promoted malignant phenotypes in PCa cells and HUVEC tube formation. **A**, **B** The overexpression efficiency of LRG1 after transfection with targeted LRG1 (PCDH-LRG1) or negative control (CTR) was confirmed by RT-qPCR and western blot in DU145, LNCaP and PC-3 cells. **C**, **D** Overexpression of LRG1 enhanced proliferation and colony formation of DU145, LNCaP and PC-3 cells, especially PC-3 and LNCaP cell lines. *p < 0.05; **p < 0.01; ***p < 0.001 (C two-way ANOVA, D Student, s t test). **E** Wound healing experiments on PC-3 and LNCaP cells showed that LRG1 overexpression significantly increased the percentage of wound healing at 0 h and 48 h in experimental groups compared with the control group. **F** The transwell experiment of PC-3 and LNCaP showed that the invasion of migratory cells was significantly enhanced in the LRG1 overexpressing group compared with the control group (Vector). **G** LNCaP overexpressed exosomes derived from cell lines may be involved in prostate cancer angiogenesis. Representative images showed HUVEC tube formation at 4 h and 8 h after treated with the same amount of exosome. *p < 0.05; **p < 0.01; ***p < 0.001 (Student, s t test)

To investigate the function of LRG1, the lentivirus constructs stably expressing PCDH-LRG1 or PCDH-control was introduced into DU145, PC3 and LNCaP using lentivirus-mediated transduction, generating LRG1 overexpressing and control cells. The efficiency of overexpression was further examined by qRT-PCR and western blot analysis (Fig. 9A, B). Next, the tumorigenicity of LRG1 was assessed by cell proliferation and colony formation assays. The results showed that ectopic LRG1 overexpression promote PCa cells growth and single cell colony formation ability in vitro (Fig. 9C, D). Meanwhile, the effect of LRG1 on cell motility was therefore studied by in vitro cell migration and invasion transwell assays. Similarly, high expression of LRG1 enhanced cellular migration and invasion ability (Fig. 9E, F). The above results demonstrate LRG1 plays a key role in the development of PCa.

### The exosomal LRG1 promoted the process of HUVEC tube formation

The most important function of LRG1 is to participate in the formation of pathological angiogenesis and the loss of perivascular cells to cause vascular instability [24]. Due to the role of exosome-derived LRG1 protein in prostate cancer angiogenesis is unknown. Therefore, tube formation assay was used to access the angiogenesis ability by PCa cell line derived LRG1 exosomes. Exosome-depleted FBS was obtained by Ultracentrifugation, which was further used for LNCaP culture. After 48 h cell culture, the supernatant of LNCaP was collected and ultracentrifuged for exosome isolation. HUVEC cells were pretreated with exosomes for 24 h with LRG1 secreted by LNCaP control or LNCaP-LRG1 overexpression cells. Results showed that LRG1-overexpressed cell derived exosomes promoted tube formation of HUVEC cells than that from control cells, indicating that exosomal LRG1 protein may be involved in the process of PCa angiogenesis, which facilitate the distant metastasis of advanced prostate cancer (Fig. 9G).

## Conclusions

The progression of PCa patients to CRPC varies widely among individuals. At present, there is still a lack of highly specific and sensitive molecular markers in clinical practice to predict the progression of PCa to CRPC and evaluate the therapeutic effect. Exosomes have provided a new direction for the precise diagnosis and treatment of malignancies in recent years for their natural advantage in liquid biopsy and therapeutic carrier. Urine is a very advantageous source of liquid biopsy markers because of its simple and easy access. Prostate cancer urine markers have been developed into commercial qualitative detective products. *e.g.* PSA3 is the first FDA-approved urine RNA-marker, and ExoDx kit is based on three urinary exosome-derived gene (PCA3, ERG and SPDEF) combination [25]*.* Howerver, urine markers are also easily affected by urine volume, concentration and the presence of other substances. In terms of quantitative monitoring markers, blood is relatively more stable, and plasma-derived exosome markers have more advantage for early prediction and efficacy monitoring of CRPC.

In the present study, we collected plasma from TFCs and PCa and CRPC patients, and isolated and purified exosomes for proteomic analysis. Our data indicated that in terms of exosome abundance, there were no significant differences between various disease groups as a whole, but individual variations observed. Since we used 0.22-µm filtration, most of the larger vesicles were directly removed. Thus, good homogeneity was observed in all of the samples. In the comparison of PCa patient and TFCs, we identified 27 DEPs, including 18 upregulated and 9 downregulated proteins. The expression levels of LRG1, C7, SHBG, HRG, SERPINF1, and LUM in the PCa group were twofold or higher than those in the TFC group. Conversely, the expression levels of ExtL2, UGP2, RSU1, TUBB1, HBD, PF4, HbA1, C1R, and HBB were reduced by > 50% in comparison with those in the TFCs. We also observed APOE (a low-density lipoprotein that transport cholesterol from peripheral tissues to the liver for metabolism) in which group? was 1.7-fold higher than that in the TFC group and the AUC curve of APOE was 0.734 for PCa classification (Additional file 1: Fig. S2A–C). Although exosomal APOE were reported as potential biomarkers in several studies, we still need to be very cautious to verify if its exosome resource, as the molecular size of low-density lipoproteins is very close to exosomes, and APOE is also a high accumulative plasma protein [26].Two exosomal-protein LRG1 and ITH3 and their combination showed a good potential as liquid biopsy markers to distinguish CRPC from PCa. Current data based on a cross-sectional study which recruits TFC, PCa, and CRPC patiets. For validation of the values of LRG1 and ITH3 or their combination as predictive markers for early prediction and monitoring of CRPC, a longitudinal cohort study with the observation of their levels during the whole natural history of CRPC progression need further investigation.

LRG1 is a member of the leucine-rich repeat sequence (LRR) protein family with eight repeat sequences. Previous studies have demonstrated that LRG1 is involved in the progression of tumors by promoting angiogenesis, including pancreatic cancer, lung cancer, bladder cancer, and colon cancer [27–30]. The association of abnormal increase of plasma LRG1 with the degree of PCa malignancy was observed [31]. Wang et al. showed that LRG1 is indispensable for promoting mouse ocular angiogenesis, and the lack of LRG1 was associated with significant pathological ocular angiogenesis through dysregulation of the TGF-β signaling pathway [24]. In our study, LRG1 was enriched in CRPC, whose level was 1.7 times higher than that in PCa group in PRM validation, compared to a two-fold elevation in untargeted proteomics. However, whether the exosomal LRG1 derived from prostate cancer cells and the functional role of LRG1 protein in prostate cancer is far from known. IHC examination showed that LRG1 protein was significantly upregulated in advanced prostate cancer and functional assay revealed that ectopic expression of LRG1 can significantly enhance the malignant phenotype of prostate cancer cells. More importantly, PCa cell derived LRG1-overexpressed exosomes remarkably promoted angiogenesis. However, if LRG1 plays a role in prostate cancer distant metastasis and the machemism of LRG1 induced angiogenesis needs further study.

ITIH3 belongs to the α-trypsin inhibitor family and is enriched in the extracellular matrix and blood [32]. One known function of this protein family is covalent binding to hyaluronic acid (HA) to stabilize the extracellular matrix (ECM). Several studies have suggested that ITIH3 exerts a tumor suppressor role in disease progression. For example, low expression of ITIH1 and ITIH3 resulted in a low number of lung metastases in a xenograft mouse model and increased the ability of cell attachment in vitro [33]. In addition, Hamm et al. demonstrated that frequent loss of ITIH3 was observed in many solid tumors, such as lung cancer, gastric cancer, breast cancer, and ovarian cancer. Conversely, a significant increase in ITIH3 expression was observed in the plasma of patients with lung cancer [34, 35]. In our study, we observed that the level of ITIH3 derived from CRPC patients was 2.04-fold higher than that in the PCa group. We speculated that ITIH3 distribution varied between the inside and outside of cells or even exosomes, which is likely due to ADT. Whether ITIH3 enrichment is specific to ADT requires future studies.

For untargeted metabolomics study, a total of 206 secondary spectrograms were obtained by mass spectrometry. In the comparison of PCa patients versus TFC, two elevated metabolites were observed in PCa samples relative to the TFC group, 2-(2-methylbutanoyl), and acetylglycine. Moreover, creatinine, dihydrothymine, and hydroxyoctanoic acid were higher in the TFC group, whose levels were 2–2.5 times than those in the PCa group. Interestingly, acetylglycine belongs to the amino acid pathway and may be involved in immunoregulation [36, 37]. There was also some evidence that acetylglycine serves as a biomarker for disease diagnosis. Jonsson et al. reported that a significant increase in the level of acetylglycine in plasma was observed in glioma patients [38]. Another example is the use of a combination of urinary acetylglycine and gamma-glutamylalanine to identify Vogt-Koyanagi-Harada disease, which is a multisystem disease of presumed autoimmune cause. Another metabolite, dihydrothymine, is an intermediate metabolite of thymine. Aberrant elevation of dihydrothymine may induce cytotoxicity. One interesting example illustrated that dihydropyrimidine dehydrogenase (DPYD) was induced by EMT-promoting transcription factors and generated dihydrothymine, which is necessary for the EMT process [39]. However, different results were observed in our study, in which low levels of dihydrothymine were enriched in PCa samples. One potential explanation for the reduced dihydrothymine expression is that PCa may prefer to maintain high levels of dihydrothymine in internal tumor cells rather than to release them as exosomes.

Comparisons between CRPC and PCa showed that the cycloartocarpin and 2-methylglutaric acid content in the CRPC group were more than twofold those in the PCa group, while the levels of tridecanoic acid, undecanoic acid, and hydroxyoctanoic acid were abundant in the PCa group and 2–2.8-fold higher than those in the CRPC group. 2-Methylglutaric acid is an alpha, omega-dicarboxylic acid. Metribolone (R1881), also known as methyltrienolone, is a synthetic and orally active anabolic–androgenic steroid (AAS) that is widely used in scientific research as a ligand of interest in the androgen receptor (AR). Putluri et al. used 10 nM synthetic androgen (R1881) to treat VCaP prostate cancer cells for 24 h and found that 2-methylglutaric acid levels were significantly elevated in comparison with the levels in untreated controls [40]. Thus, 2-methylglutaric acid could be a downstream metabolite dependent on AR activation. In our study, the 2-methylglutaric acid level was only increased in the CRPC group, which may suggest that relatively high levels of 2-methylglutaric acid are associated with ADT resistance.

Other differential metabolites included cycloartocarpin, tridecanoic acid, undecanoic acid, and hydroxyoctanoic acid. There is no strong evidence for the involvement of these metabolites in tumor progression and development, and they are unlikely to be the byproduct of CRPC. However, these metabolites exhibited excellent performance in distinguishing CRPC. ROC curve analysis was also performed for a series of metabolites, including cycloartocarpin, 2-methylglutaric acid, and hydroxyoctanoic acid, and the results showed that their AUC values were 0.87, 0.86, and 0.88, respectively. Subsequently, we constructed a combination diagnosis model using these four metabolites. Surprisingly, the AUC value of this model was 0.97, indicating an excellent ability to differentiate PCa from CRPC.

In summary, our current study applied an integrated proteomics and metabolomics analysis to describe the protein and metabolic profiles of plasma exosomes from CRPC and PCa patients as well as TFC control cohort. Several exosomal proteins and metabolites and their combinations showed potential values as CRPC markers that facilitate the discrimination of CRPC from PCa and TFC patients. Functional study of exosomal protein LRG1 confirmed its important role in PCa malignant progression.

## Supplementary Information

**Additional file 1: Fig. S1.** The GO enrichment analysis based on between comparisons of PCa/TFC and CRPC/PCa.** Fig. S2.** Bar graphs summarize the quantification of APOE levels in each group and ROC curve analysis (A) Untargeted proteomics exhibited the relative quantification of APOE. (B) PRM Validation of APOE by independent cohort TFC=21, PCa=15 and CRPC=12. (C) ROC curve showed the overall performance of the classifier. (D-E) Correlation analysis of APOE as well as metabolites.

## Data Availability

All data generated or analyzed during this study are included in this published article.

## Abbreviations

LRG1: Leucine-rich alpha2-glycoprotein 1
ITIH3: Inter-alpha-trypsin inhibitor heavy chain H3
CRPC: Castration resistant prostate cancer
LC–MS: Liquid chromatography–mass spectroscopy
NFX1: Phosphorylated nuclear transcription factor X box binding 1
PRM: Targeted 4D-parallel reaction monitoring
ROC: Receiver operating characteristic
ADT: Androgendeprivation therapy
PSA: Prostate-specific antigen
CA: Carbohydrate antigen
UPLC: Ultra-performance liquid chromatography
HUVEC: Human umbilical vein endothelial cell
TEM: Transmission electron microscopy
NTA: Nanoparticle tracking analysis
GO: Gene ontology
OPLS-DA: Orthogonal projections to latent structures-discriminant analysis
TMA: Tissue microarray
