# Supplementary material for: Alterations of plasma exosomal proteins and motabolies are associated with the progression of castration-resistant prostate cancer
Source: J Transl Med. 2023 Jan 21;21:40. doi: 10.1186/s12967-022-03860-3 (PMC9867857; doi:10.1186/s12967-022-03860-3)

Suplemental Figure 1

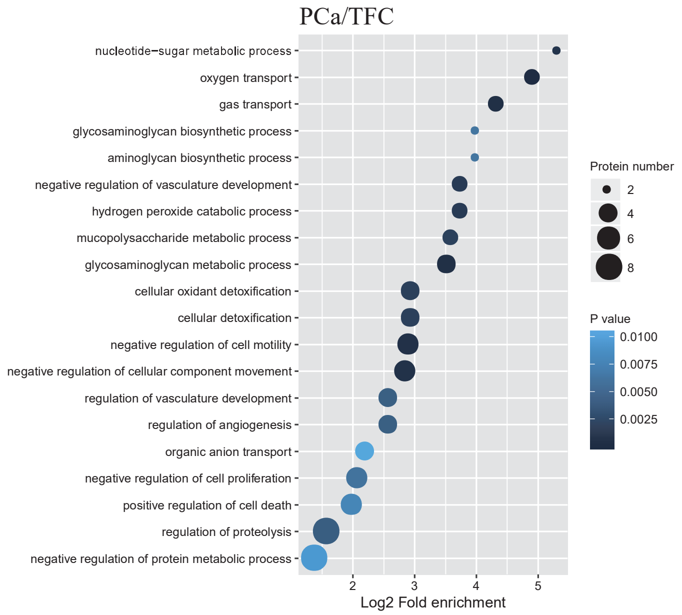

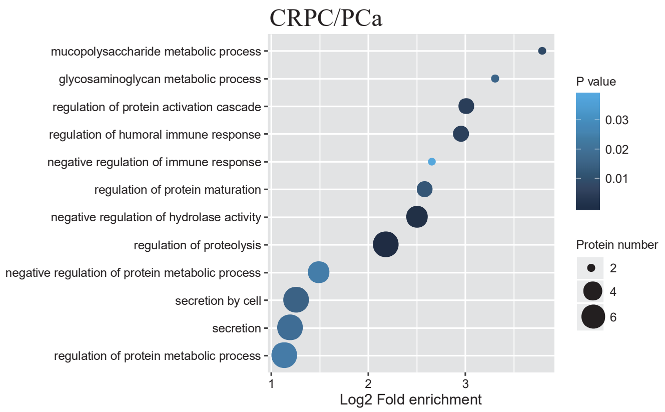

Supplementary Fig. 1. The GO enrichment analysis based on between comparisons PCa/TFC and CRPC/PCa.

Supplemental Figure 2

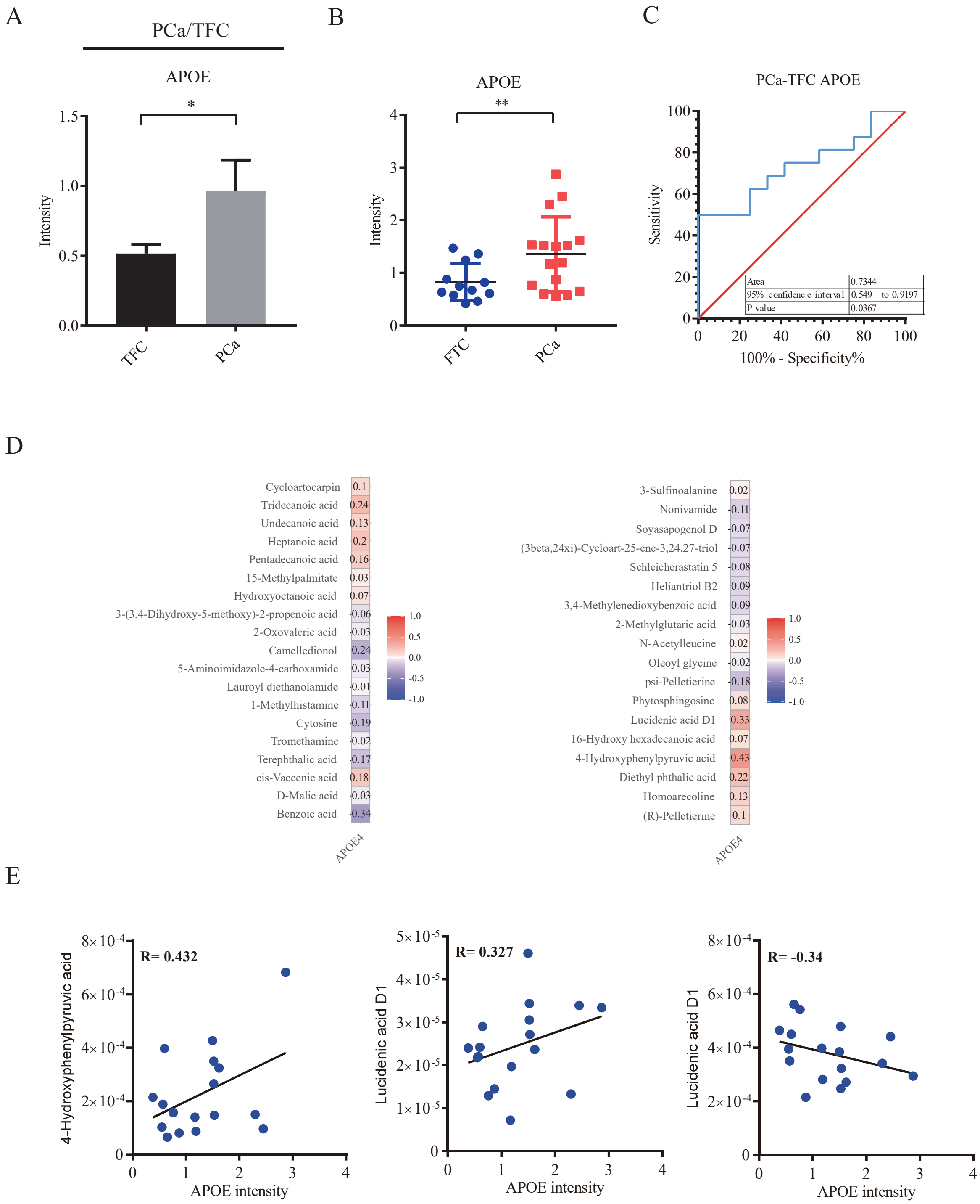

Supplement: Supplementary file 1 — Additional file 1: Fig. S1. The GO enrichment analysis based on between comparisons of PCa/TFC and CRPC/PCa. Fig. S2. Bar graphs summarize the quantification of APOE levels in each group and ROC curve analysis (A) Untargeted proteomics exhibited the relative quantification of APOE. (B) PRM Validation of APOE by independent cohort TFC=21, PCa=15 and CRPC=12. (C) ROC curve showed the overall performance of the classifier. (D-E) Correlation analysis of APOE as well as metabolites. [file 12967_2022_3860_MOESM1_ESM.pdf]
